# Searching for the proverbial needle in a haystack: advances in mosquito-borne arbovirus surveillance

**DOI:** 10.1186/s13071-018-2901-x

**Published:** 2018-05-29

**Authors:** Ana L. Ramírez, Andrew F. van den Hurk, Dagmar B. Meyer, Scott A. Ritchie

**Affiliations:** 1 College of Public Health, Medical and Veterinary Sciences, James Cook University, PO Box 6811, Cairns, QLD 4870 Australia; 2 Astralian Institute of Tropical Health and Medicine, James Cook University, PO Box 6811, Cairns, QLD 4870 Australia; 3 Public Health Virology, Forensic and Scientific Services, Department of Health, Queensland Government, Coopers Plains, QLD 4108 Australia

**Keywords:** Arboviruses, Surveillance, Mosquito, Sentinel animals, Honey-based surveillance, Next-generation sequencing

## Abstract

Surveillance is critical for the prevention and control of mosquito-borne arboviruses. Detection of elevated or emergent virus activity serves as a warning system to implement appropriate actions to reduce outbreaks. Traditionally, surveillance of arboviruses has relied on the detection of specific antibodies in sentinel animals and/or detection of viruses in pools of mosquitoes collected using a variety of sampling methods. These methods, although immensely useful, have limitations, including the need for a cold chain for sample transport, cross-reactivity between related viruses in serological assays, the requirement for specialized equipment or infrastructure, and overall expense. Advances have recently been made on developing new strategies for arbovirus surveillance. These strategies include sugar-based surveillance, whereby mosquitoes are collected in purpose-built traps and allowed to expectorate on nucleic acid preservation cards which are submitted for virus detection. New diagnostic approaches, such as next-generation sequencing, have the potential to expand the genetic information obtained from samples and aid in virus discovery. Here, we review the advancement of arbovirus surveillance systems over the past decade. Some of the novel approaches presented here have already been validated and are currently being integrated into surveillance programs. Other strategies are still at the experimental stage, and their feasibility in the field is yet to be evaluated.

## Background

Arthropod-borne viruses (arboviruses) transmitted by mosquitoes are of public health and veterinary importance globally causing disease syndromes including encephalitis, viral haemorrhagic disease and arthritis. Dengue viruses (DENVs) alone cause an estimated 96 million clinical cases a year, especially in the tropics and sub-tropics [1]. The flaviviruses, Japanese encephalitis virus (JEV) and West Nile virus (WNV), are major causes of viral encephalitis throughout their geographical range. Recently, the expansion of chikungunya (CHIKV) [2] and Zika (ZIKV) [3] viruses in the Western Hemisphere, and the yellow fever (YFV) outbreaks in Africa [4] and Brazil [5] have highlighted the continuing threat emerging and re-emerging arboviruses pose.

**Fig. 1:**
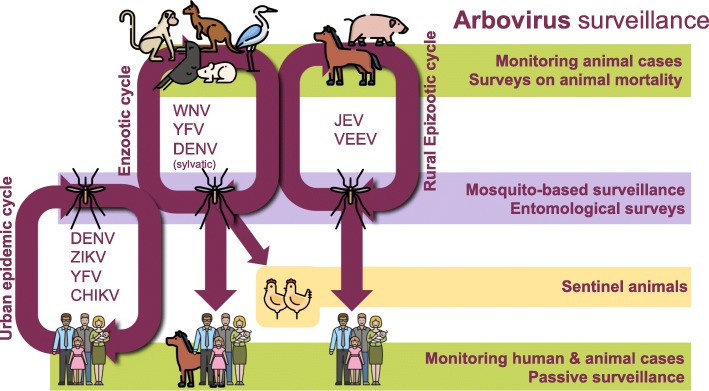
Transmission cycles of arboviruses and different strategies for arbovirus surveillance

With the exception of YFV [6] and JEV [7], there are currently few vaccines or antiviral drugs available against most of these viruses. Thus, prevention and control of most arboviruses is almost solely reliant on effective mosquito management. This can be enhanced by surveillance, where detection of elevated or emergent virus activity serves as a warning system to implement appropriate actions to reduce the severity and duration of outbreaks. However, designing an appropriate arbovirus surveillance system is challenging. Arboviruses have complex transmission cycles with dual-host tropism: they replicate in vertebrate hosts (such as birds or mammals) and arthropod hematophagous vectors (such as mosquitoes or ticks) [8]. This complexity needs to be accounted for, and an ideal surveillance system should rely on different sources of information (Fig. 1), and can include meteorological data, evidence of virus infection in vertebrate hosts, entomological surveys, virus detection in vectors, and reports of human or animal disease. The scale of surveillance can vary regionally [9] and is particularly challenging in remote locations, or in areas with limited resources and infrastructure.

Given the broadness of this subject, few attempts have been made to provide a synthesis of arbovirus surveillance methods. The objective of this review is to describe the development and implementation of mosquito-borne arbovirus surveillance strategies. First, we evaluate traditional methods that have been commonly used where arboviruses are a public health threat, then outline and assess recently developed methodologies, before identifying future research needs.

## Methods for arbovirus surveillance

### Monitoring human and animal disease

Human or animal case surveillance relies on hospitals, laboratories and health practitioners notifying public health authorities of confirmed or suspected cases of arbovirus infection that occur in the population. Almost every state in the United States conducts surveillance of human WNV cases as a part of the national arbovirus surveillance system, ArboNET [10], whilst in Australia, human arbovirus disease notifications are monitored using the National Notifiable Disease Surveillance System (NNDSS) [11]. These surveillance systems require strict case definitions and laboratory diagnostic testing criteria, as well as demographic, clinical, laboratory and epidemiological information [12]. In the summer and autumn of 1999, reports of dead crows played a critical role in identifying the outbreak of WNV in New York [13]. With bird cases often preceding human cases by up to 3 months, it served as an ideal early warning system for WNV [14]. In Argentina [15] and Brazil [16], dead howler monkeys acted as an early warning for sylvatic transmission of YFV and prompted vaccination campaigns in the human population in 2008 and 2017, respectively.

A major limitation of monitoring human and animal cases is that confirmatory laboratory testing is not available in many limited resource countries, so arboviral disease is diagnosed on clinical symptoms. However, symptoms can overlap between arboviruses, as well as with non-arbovirus pathogens, complicating their clinical diagnosis. Furthermore, most arbovirus infections are mild, or sub-clinical, which may lead to them being under-reported. Ultimately, using human and animal case data is not ideal, since it indicates that active transmission is already occurring.

### Vertebrate host arbovirus surveillance: sentinel animals

Sentinel animals provide evidence of virus activity and increased risk to the target animal or human population [17]. For this, immunologically naïve animals are deployed in a specific location, bled on a defined schedule, and tested for the presence of virus-specific antibodies as an indication of exposure. Virus isolation or molecular detection on pre-seroconversion blood samples can provide an isolate and/or a sequence for genotypic analysis of circulating virus strains [18]. A suitable sentinel animal should: (i) be susceptible to the monitored virus; (ii) develop an antibody response that can be detected in serological assays; (iii) have low morbidity and mortality; (iv) be attractive to the vector; (v) be easy to handle; and (vi) allow for multiple sampling [12].

Different vertebrate species are used as sentinels (Table 1) and choice of animal is dependent on the target virus. In terms of WNV surveillance, some studies have suggested that the use of sentinel chickens is the most sensitive indicator of virus activity, when compared with other methods, such as detection of seroconversion in wild birds and virus isolation from mosquito pools [19]. Whilst they can undoubtedly serve as an early warning system, in some areas of the USA, sentinel chickens to monitor WNV have proven unsuccessful, since seroconversions were detected only after the onset of human cases [20, 21].

**Table 1:** Animal species that have been used as sentinels for arbovirus surveillance

Animal	Virus	Example location	References
Chickens	WNV	USA, UK	[119, 120]
	SLEV	USA	[121]
	MVEV, WNV_KUN_	Australia	[122]
Pheasants	WNV, SLEV, EEEV	USA	[123, 124]
Pigs	JEV	Japan, Australia, Thailand	[125–127]
Dogs	WNV	USA, Africa	[128, 129]
	JEV	Japan, Thailand	[130, 131]
Sheep and goats	RVFV	Africa, Saudi Arabia	[132–134]
Cattle	BTV, Akabane	Australia, Papua New Guinea, Japan	[135–137]
Horses	EEEV, WEEV	Argentina	[138]
	WNV, SLE	Colombia	[139]
Hamsters	EEEV, VEEV	USA, Central and South America	[140–143]
Non-human primates	YFV	Brazil, Argentina	[144–147]

*Abbreviations*: WNV, West Nile virus; SLEV, St. Louis encephalitis virus; MVEV, Murray Valley encephalitis virus; WNV_KUN_, West Nile virus (Kunjin subtype); EEEV, eastern equine encephalitis virus; JEV, Japanese encephalitis virus; RVFV, Rift Valley fever virus; BTV, bluetongue virus; WEEV, western equine encephalitis virus; VEEV, Venezuelan equine encephalitis virus; YFV, yellow fever virus

Even though sentinel animal surveillance enables the timely detection of circulating arboviruses, it also comes with limitations. In many cases, the locations of enzootic arbovirus foci are unknown or difficult to access. Thus, animals are placed near towns, which may be too far from virus foci to detect elevated activity [22]. Furthermore, some animals serve as amplifying hosts (i.e. pigs for JEV) increasing the risk of transmission to humans [23]. Additionally, the cost of rearing and replacing sentinel animals, especially in remote locations, can be prohibitive [24, 25], and bleeding large animals presents a workplace health and safety hazard [26]. There are also ethical considerations associated with the use of sentinel animals [27]. Finally, closely related viruses (i.e. JEV, WNV and Murray Valley encephalitis virus (MVEV)) can cross-react in some serological assays, requiring confirmation by other methods to obtain unequivocal results [28].

Another approach to vertebrate host surveillance relies on monitoring wild vertebrates or livestock, which are captured, sampled and released [12]. However, one of the biggest issues with surveillance of these animals is the cross-reaction between antibodies and the interpretation of the results. Given that many of these animals are mobile, it is difficult to determine exactly when and where an animal acquired the infection, especially since IgG antibodies are present for the life of the animal.

### Mosquito-based arbovirus surveillance

Mosquito-based arbovirus surveillance monitors vector populations and virus infection prevalence within them. Mosquitoes are collected, identified, pooled by species or other taxonomic grouping, and sent to the laboratory where they are tested for virus infection status. There are different strategies for mosquito collection. In areas with low-level mosquito infections or early in the transmission season, efforts should be directed towards performing targeted surveillance at “hotspots” where a high likelihood of arbovirus presence is suspected; as vector populations increase later in the season, the number of sampling sites should be expanded for broader monitoring [29]. There are a variety of commercial traps designed to collect mosquitoes, the design of which and application have been comprehensively reviewed elsewhere [30, 31]. It is essential that the selection of the collection method takes into consideration the physiological and behavioural characteristics of the studied vector [32] (Table 2).

**Table 2:** Collection methods commonly used for mosquito-based arbovirus surveillance

Mosquito behaviour	Collection method	Advantages	Disadvantages	References
Host seeking	Human-landing catches^a^	Larger collections than resting or oviposition collections. Collections can be increased by using CO_2_ or chemical lures	Most traps require batteries or AC power to operate. Depending on environmental conditions, the fan components are prone to malfunction. Require CO_2_ as the primary attractant	[148]
	BG Sentinel			[149]
	CDC-light trap			[150]
	EVS-trap			[151]
	Mosquito Magnet™			[152]
	Animal baited traps			[153–155]
Resting	CDC-backpack aspirator	More blood fed mosquitoes collected, ideal for blood meal analysis	Labour intensive and inefficient mosquito capture	[156]
	Prokopack			[157]
	Resting boxes			[158–160]
Oviposition	Sticky ovitraps	Mosquitoes have bloodfed and thus a higher probability of detecting positive mosquitoes. Targets *Aedes-*borne viruses such as DENV and CHIKV	Smaller collections than other methods, thus all mosquitoes can be easily processed	[161–163]
	Gravid *Aedes* trap (GAT)			[66]
	CDC-gravid trap			[164]

^a^Although this method has been used for arbovirus studies in the past, it has considerable drawbacks, including the risk of infection to the collector, which is considered unethical even illegal in some countries

*Abbreviations*: CDC, Centers for Disease Control and Prevention; EVS, Encephalitis virus surveillance

A variety of methods have been utilized for detection of arboviruses in captured mosquitoes. Historically, arbovirus isolations were conducted in animals, such as suckling mice and chickens. With the development and establishment of cell lines, virus isolation in cell culture became the gold standard for arbovirus detection from pools of mosquitoes. This method can only detect viable viruses, so a cold chain keeping samples at ultralow temperatures during transport needs to be maintained to preserve virus infectivity [33]. Maintenance of a cold chain requires the use of dry ice or liquid nitrogen shippers in the field, which can be logistically challenging. Virus isolation is time consuming and obtaining definitive results can take weeks, which defeats the purpose of using it for early warning. Some viruses do not replicate on common cell lines used in the laboratory. This can be the case for previously unrecognized or unknown viruses, such as insect-specific flaviviruses (ISF) that do not grow in vertebrate cells [34]. Virus isolation can be expensive and requires special infrastructure and trained personnel. However, even with these limitations, virus isolation is still an important method for arbovirus diagnostics, as it increases viral titer, which allows for full genome sequencing and provides viruses for phenotypic characterization.

Nucleic acid detection using RT-PCR has become one of the most popular methods of virus detection and has potentially displaced virus isolation as the new gold standard. Real time quantitative RT-PCR (qRT-PCR) platforms, such as TaqMan®, are ideal for routine testing of mosquitoes, since they reduce processing time significantly (sometimes to less than an hour), allowing for high throughput screening [35, 36]. Since these assays detect both infectious virus and RNA, they have comparable or better sensitivity than virus isolation [37]. Depending on the protocol or application, these techniques enable the detection of one infected individual from a pool of up to 5000 non-infected mosquitoes [38, 39]. Additionally, although a cold chain is still recommended, it has been possible to detect viral RNA from dead mosquitoes kept for several weeks in hot and humid conditions by qRT-PCR [33, 39, 40]. Currently, a variety of qRT-PCR assays exist for the detection of almost every arbovirus of human (and veterinary) importance, with some even available in multiplex format [41]. In spite of this, it is important to note that RT-PCR and qRT-PCR will only pick up RNA from viruses that the primers and probes were designed to detect [42]. Historically, one of the main drawbacks of this method has been its high installation and reagent costs, limiting its use in low-resource settings. However, recently, costs associated with qRT-PCR have dropped considerably making it an accessible alternative for routine screening**.**

Rapid antigen detection assays were initially developed to test clinical samples but have proven to be a useful tool to test mosquito pools in the field [43]. These assays allow for qualitative detection of arboviruses, and have the advantage of being rapid, without the need for specialized equipment. Currently, there are tests commercially available for a variety of viruses including CHIKV [44], DENV [45] and WNV [43], among others. In Singapore [46] and Malaysia [47], a dengue NS1 rapid test has been used to detect infected mosquitoes as part of a routine surveillance programme. These tests have shown high specificity for the target virus, although some assays have reduced sensitivity when compared with molecular methods [48, 49]. However, although they may provide an underestimate of infection rate, they provide a first screen and have applicability in regions without access to more resource intensive or expensive diagnostic capacity.

Traditional mosquito-based surveillance systems that target processing of pools of mosquitoes come with inherent limitations. Mosquito populations often have very low carriage rates, whereby only one in 1000 mosquitoes is actually infected [50]. To increase the probability of detection, large numbers of mosquitoes are required, resulting in numerous mosquitoes to identify, pool and test, increasing laboratory costs and turnaround time. Additionally, many traps require attractants, such as CO_2_, to increase collections. This comes in the form of dry ice or pressurized cylinders, which may not be readily available, or only allow overnight deployment of the trap. A cold chain of storage at < -50 °C is required to preserve the integrity of the virus for detection, which can be a challenge in remote locations. Finally, specialized laboratory equipment and infrastructure is required for diagnostics, which might not be available in developing countries.

### Novel methods for arbovirus surveillance

The majority of mosquito species feed on carbohydrates (i.e. flower nectar, honeydew or rotting fruit) which are the primary energy source of their diet [51]. One exception is *Ae. aegypti*, which appears to obtain enough energy from blood and rarely feeds on sugar in domestic environments [52]. The ingestion of carbohydrates is important for the survival of the mosquito, and plays an indirect role in disease transmission, allowing an infected female to live long enough to become infective [53]. It was hypothesized by Doggett et al. [54] and confirmed by van den Hurk et al. [55], that infected mosquitoes expectorate virus while sugar feeding, which can be detected using molecular assays. This finding led to the development of novel sugar-based approaches for the detection of arboviruses in mosquitoes in the field. This system integrates purpose-built CO_2_-baited box traps, which house nucleic acid preservation cards (Flinders Technology Associates, FTA® cards) soaked in honey and on which mosquitoes feed and expectorate onto [56]. The FTA® cards inactivate any expectorated viruses and preserve the liberated RNA. The cards are then sent to the laboratory in the post without requirement of a cold-chain, where they are screened for viruses using molecular assays.

Commonly used traps employed to collect mosquitoes (i.e. CDC-light trap and Encephalitis Virus Surveillance, EVS, trap) require batteries to operate which can be logistically challenging. To circumvent this limitation, a non-powered CO_2_-baited passive box trap (PBT) was developed by Ritchie et al. [57] to collect and house mosquitoes. A variation of the PBT, the sentinel mosquito arbovirus capture kit (SMACK) was developed to increase mosquito survivorship and consequently increase the probability of infected mosquitoes feeding on the FTA® card [58]. Although designed for weekly or fortnightly servicing, the SMACK has demonstrated similar trap efficacy to the CDC-light trap and EVS trap in overnight collections, making it an alternative to traps that require batteries to operate.

Free-standing sugar bait stations have the potential to be used instead of CO_2_-baited traps [59]. These stations consist of a dental wick soaked in sucrose solution and a floral lure, such as phenyl acetaldehyde. Mosquitoes lured to the station feed on the wick, which is tested for expectorated viral RNA. The sugar bait stations do not require CO_2_ or electricity, so a number of stations can be deployed simultaneously, thus increasing geographical coverage. In a proof of concept, the sugar bait stations detected WNV before sentinel animals seroconverted in California. However, this method appears more efficacious in arid habitats, probably because of lack of competition with other sucrose sources, such as floral nectars. As sugar bait stations facilitate increased geographical coverage, they may have higher costs associated with analysing an increased number of samples, although this would be offset by savings by not having to use CO_2_ baited light traps.

Sugar-based surveillance has several advantages over traditional methods. When mosquito populations are elevated, sorting becomes time consuming, and a high number of pools can overwhelm laboratory capacity. When combined, these issues can reduce the ability to provide results in a timely manner. Sugar-based methods potentially overcome these issues, since only 1-2 FTA® cards per trap are tested, compared to a variable number of mosquito pools. As only transmitting mosquitoes will yield positive results, the presence of virus in saliva expectorate is a better estimate of transmission risk. FTA® cards can preserve viral RNA for up to 28 days [56], making this an ideal alternative for surveillance in remote or difficult to access locations, where regular servicing of traps is not feasible. Results suggest that sugar-based surveillance is a more sensitive indicator of arbovirus activity than sentinel animals. In northern Australia, it has been possible to detect WNV_KUN_ before sentinel animal seroconversions [60]. However, a comparison of the sugar-based surveillance system with existing strategies still needs to be thoroughly evaluated. Sugar-based surveillance, using either SMACK or EVS traps, has been successfully incorporated into existing surveillance programs in Australia, with multiple detections of MVEV, WNV_KUNV_, RRV, BFV, Edge Hill virus and Stratford virus [61–64].

Honey-soaked FTA® cards have the potential to be integrated into surveillance of *Ae. aegypti*-borne arboviruses. The cards have been used in Biogents sentinel traps (BGS traps) and modified double sticky ovitraps for the detection of CHIKV in French Guiana [65]. The approach appeared time consuming with only one CHIKV positive FTA® card out of 234 analysed. Traps that are more efficient at collecting *Ae. aegypti* may be able to increase trap collections, thus increasing the likelihood of detecting virus. For instance, the Gravid *Aedes* Trap (GAT) [66] collects 2.4 times more *Ae. aegypti* and significantly more gravid females than double sticky ovitraps [67], which could increase the chances of finding positive mosquitoes. However, *Ae. aegypti* collections are usually small, and in many cases, it would be easier to pool the mosquitoes (or alternatively, squash them into FTA® cards [68]) and process them by molecular methods.

Like any system, sugar-based surveillance has some limitations. Perhaps its main limitation is that the cycle threshold (C_t_) values obtained by real time RT-PCR are high (> 30 cycles), reflecting the relatively small amount of saliva expectorated by mosquitoes [69]. Additionally, this method will only detect positive mosquitoes after the extrinsic incubation period which, depending on the virus, can last from two to 14 days. Thus, the proportion of mosquitoes in a population that survive to transmit the virus can be quite low. In order to increase mosquitoes feeding on the FTA® cards, trapped mosquitoes must be kept alive in the trap for as long as possible. The SMACK was developed to include a water reservoir in the trap to increase humidity, the lack of which can be a problem in remote and arid locations. To save on reagent costs, some agencies will wait until they have sufficient samples to batch together, which can extend the turnaround time. Finally, sugar-based surveillance does not provide data on the mosquito species that expectorated the virus. Instead, detection of virus on a FTA® removed from a trap could be used to trigger intensive trapping to collect mosquitoes for pooling and processing to provide information on potential vectors at a given time point or location.

A potential way to increase sensitivity of sugar-based surveillance systems is through the collection and analysis of mosquito excreta. When mosquitoes feed on a sucrose solution it takes approximately 30 min for it to reach the midgut, after which excreta is ejected from the anus [70]. In terms of pathogen detection, the focus has mainly been on the detection of filarial nematodes, such as *Brugia malayi* [71] and *Plasmodium vivax* [72]. In the late 1920s, de Beaurepaire Aragão and da Costa Lima performed a series of experiments in which they infected rhesus macaques with the excreta collected from YFV infected *Ae. aegypti* [73–75]. Laboratory-based experiments have recently demonstrated that *Ae. aegypti* with a disseminated infection excrete DENV RNA, which can be detected through qRT-PCR [76]. The rate of detection was higher in excreta samples, 89%, compared with 33% for saliva samples. This suggests that collection of excreta from trapped mosquitoes could enhance the sensitivity of current sugar-based surveillance systems. This is not surprising, given that mosquitoes excrete considerably more fluid than they salivate (~1.5 μl [77] *vs* 4.7 nl [69]). Integration of excreta collection into current surveillance systems would require modification of current trap designs to selectively capture mosquito excreta.

## Advances in arbovirus detection, characterization and data interpretation

### Next-generation sequencing for the detection of arboviruses

Traditionally, diagnostic assays utilised in arbovirus surveillance programs only screen for characterised endemic and enzootic viruses. Because virus specific primers and probes are used for molecular diagnostics, it is likely that many other viruses, whether pathogenic or not, remain undetected. Metagenomic analysis using next-generation sequencing (NGS), allows for the simultaneous identification of viruses, mosquito species, and endosymbionts, such as *Wolbachia*, from a single mosquito in a single reaction [78] without prior sequence knowledge. This approach relies on bioinformatics tools to analyse the millions of sequence reads [79–81] and the availability of high-quality sequence databases to analyse the large and complex datasets generated. In Australia, viral metagenomics has been used for the identification of multiple arboviruses, including novel rhabdoviruses, bunyaviruses [82] and mesoniviruses [83] from field collected mosquitoes.

At this stage, NGS methods have some disadvantages compared with other molecular methods of virus detection. NGS is less sensitive than qRT-PCR for the detection of samples with low virus titres [84]. At present, the costs associated with NGS are higher than the cost of qRT-PCR, and its associated equipment has a relatively large laboratory footprint. It also requires intimate bioinformatics knowledge and reference sequence databases to analyse the data produced. Over the past years, there has been advancement in the hardware used for NGS, with equipment getting smaller and cheaper. The first hand-held portable sequencer (MinION) is already available on the market. This platform reduces processing time significantly (e.g. < 6 hours for detection of CHIKV from blood samples [85]). Even with operational challenges, the MinION’s high portability and low energy requirements have enabled its use in extreme field conditions [86] and it has been used to investigate outbreaks of Ebola [87] and *Salmonella* [88]. It has recently been demonstrated that the MinION can be used for metagenomic arbovirus detection from infected mosquitoes [89], so it could be used during arbovirus outbreaks. Although the MinION still has limitations, such as high error rates and requirement for an internet connection for base calling, technologies like this, together with lower reagent costs, will be crucial in making sequencing accessible in the field in the near future.

### Xenosurveillance

Mosquitoes have the potential to act as environmental samplers (“biological syringes”) that feed on the blood of a variety of vertebrate hosts. Xenosurveillance offers an alternative to directly sampling hosts, a process that is time consuming and requires individual informed consent in the case of humans or animal ethics approval, in the case of veterinary pathogens. Mosquitoes can be used as a proxy for syringe sampling of small animals for virus titer determination [90]. This approach has mainly been used to study vector-borne pathogens, such as filarial parasites [91] or apicomplexans [92]. For example, in Sri Lanka, xenosurveillance has been successfully used to map areas with persistent *Wuchereria bancrofti* after mass drug administration programmes [93]. Furthermore, it has been possible to detect DENV from (non-competent) *Anopheles stephensi* mosquitoes 24 h after ingestion [94]. In addition to viruses that actively replicate in them, engorged mosquitoes potentially possess viruses or other pathogens that do not replicate in them but might be present in hosts they feed upon [95]. Xenosurveillance monitors these potential non-vector borne human and animal pathogens [96] by performing nucleic acid detection or vector enabled metagenomics [97] on mosquito samples. Mosquitoes have been successfully used to monitor non-mosquito borne pathogens such H5N1 influenza virus [98], Epstein-Barr virus, canine distemper virus [96], human herpesvirus, human papillomaviruses, anelloviruses and circoviruses, among others [95].

One of the main limitations of xenosurveillance is the difficulty in collecting sufficient blood engorged mosquitoes for analysis. Some of the methods to collect engorged mosquitoes (i.e. use of an aspirator) are labour intensive and can be intrusive, especially when sampling inside houses and villages [99]. To circumvent this issue, mosquito excreta could be used to provide the template for xenosurveillance. Indeed, hepatitis B virus, which does not replicate in the vector, has been detected in mosquito excreta by RT-PCR and Southern Blot up to 7 days after the ingestion of an infectious blood meal [100].

### Emerging technology

Integration of data acquisition, storage and sharing methodologies, such as cloud networks and geographic information systems, will form an integral component of surveillance and control programmes. An example of this is the Intelligent Dengue Monitoring technology (MI-Dengue) developed in Brazil [101]. MI-Dengue consists of an array of tools to collect gravid *Ae. aegypti* females, collect field data, detect virus and create georeferenced infestation maps that are available in real time, providing information to optimize vector control. This system has been successful at reducing dengue in the municipalities that have adopted it.

In the age of mobile phones, social media and internet, citizen science will undoubtedly play an important role in disease surveillance in general. In Spain, Mosquito Alert was implemented as a system to collect reports of invasive *Ae. albopictus*. To date, it has more than 30,000 registered participants [102]. As a part of the GLOBE project sponsored by NASA, Mosquito Habitat Mapper merges data generated by citizens with satellite-based research [103]. Interestingly, with minimal training, the data generated by programmes like these is considered as reliable as data collected by experts [104]. Mobile phones, even low-end ones, can also be used as acoustic sensors to identify mosquito species [105]. All these initiatives will allow large-scale data acquisition, which is critical for adequate mosquito control.

Over the past 20 years, single device detection platforms for clinical and environmental analyses have been rapidly evolving. A promising technique for integration into surveillance programmes is the use of microfluidic devices [106] and biosensors [107] which are designed to process very small volumes of liquid, requiring minimal amount of sample and reagents to yield results in minutes [108, 109]. Some applications of these devices include diagnosis of infections caused by DENV [110–112] and CHIKV [113] from clinical samples, detection of DENV NS1 antigen from pools of mosquitoes [114] and genotyping of closely related *Anopheles* species [115].

## Conclusions

Over the past decade, there have been key scientific advances in arbovirus surveillance, particularly with regard to sample collection, virus detection and data analysis. Table 3 summarises the relative advantages and disadvantages of current and emerging surveillance methodologies. Alternative samples for virus detection, such as mosquito excreta, may enable more sensitive detection of arboviruses than existing methodologies. It has been proposed that we are on the cusp of a revolution in genomic epidemiology [116]. With NGS technologies becoming more accessible in the near future, they will enable the collection of real-time in-depth genetic information on circulating arboviruses before or during an outbreak. There is still room for improvement of surveillance systems used in remote locations where surveillance coverage is limited by cost and limited access to sites. Use of other sources of CO_2_ in mosquito traps (such as fermentation using yeast) [117] or CO_2_-free systems could provide an alternative in areas where dry ice or pressurized gas cylinders are not available. Deployment of in-field portable molecular laboratories or point of care assays could provide same-day assessment of arbovirus circulation and rapid response in these locations [118]. In the future, other technologies, such as unmanned aerial vehicles (UAVs) could be used to automate sample collection in difficult to access locations increasing the coverage of surveillance. Regardless of the surveillance system, there are always going to be issues and limitations, which can vary between jurisdictions. Currently, the extent of arbovirus surveillance varies between countries and even states with many jurisdictions lacking any form of monitoring. There is a need for sharing of arbovirus surveillance intelligence between public health agencies at regional level as a means to apply better control measures. Moreover, the implementation issues that might arise from new approaches cannot be underestimated. Agencies that are familiar with set methodologies may be reluctant to adopt new technologies or not have the capacity to implement change. Because of this, when designing new arbovirus surveillance methodologies, there should be a clear understanding of the needs and limitations of field, laboratory and public health personnel.

**Table 3:** Summary of traditional and novel arbovirus surveillance methods

Method	Advantages	Disadvantages	Application
Monitoring human and animal disease	Uses data that is already being collected by hospitals, health practitioners, and animal health personnel	Overlap of clinical symptoms within arboviruses and other pathogens. Not ideal for early warning since active transmission will be already occurring	National disease surveillance databases
Sentinel animals	Can act as an early warning system	Animals can be amplifying hosts. High costs associated with animal rearing. Cross reactivity between closely related arboviruses when using serological assays	Routine surveillance, inform control strategies
Virus isolation from pools of mosquitoes	Increases virus titer allowing for genotypic and phenotypic characterization	Time consuming. Requires special infrastructure (biological containment). Requires a cold chain	Routine surveillance, virus identification, inform control strategies
Virus detection in pools of mosquitoes using molecular assays	Allows high throughput screening. High sensitivity	Will only detect RNA from viruses that the assays were designed to detect. Requires special infrastructure	Routine surveillance, research, inform control strategies
Virus detection in pools of mosquitoes using rapid antigen detection assays	Rapid. Does not require specialized equipment. Lower cost	Lower sensitivity than molecular methods	Routine surveillance in low resource settings
Sugar-based surveillance	Does not require a cold chain. Only 1-2 samples per trap are tested potentially compared with 1000s of mosquitoes using other methods of surveillance. Better estimation of transmission risk	Relies on a nanoliter amounts of expectorate. Mosquitoes need to be kept alive for as long as possible to increase feeding on cards. Cannot be used to incriminate mosquito species as vectors. Requires special infrastructure	Routine surveillance, ideal for remote locations
Next-generation sequencing of mosquito samples	Does not require prior information (will detect any arbovirus present in the sample)	High cost. Requires bioinformatics knowledge. Requires special infrastructure	Research, virus discovery
Xenosurveillance	Mosquito acts as an environmental sampler. Allows detection of viruses that do not replicate in the mosquito	Blood engorged mosquitoes are difficult to collect	Research and surveillance of arboviruses and other pathogens

## Abbreviations

DENV: Dengue virus
JEV: Japanese encephalitis virus
WNV: West Nile virus
CHIKV: Chikungunya virus
ZIKV: Zika virus
YFV: Yellow fever virus
SLEV: St. Louis encephalitis virus
RRV: Ross River virus
BFV: Barmah Forest virus
MVEV: Murray Valley encephalitis virus
BTV: Blue tongue virus
RVFV: Rift Valley fever virus
EEEV: Eastern equine encephalitis virus
WEEV: Western equine encephalitis virus
ISF: insect-specific flaviviruses
IgG: immunoglobulin G
PCR: polymerase chain reaction
RT-PCR: reverse-transcription polymerase chain reaction
RNA: ribonucleic acid
FTA®: Flinders Associate Technologies
PBT: passive box trap
SMACK: sentinel mosquito arbovirus capture kit
C_*t*_: cycle threshold
GAT: gravid *Aedes* trap
NGS: next-generation sequencing
UAV: unmanned aerial vehicle

## References

[1] Bhatt S, Gething PW, Brady OJ, Messina JP, Farlow AW, Moyes CL (2013). The global distribution and burden of dengue. Nature..

[2] Petersen LR, Powers AM. Chikungunya: epidemiology. F1000Res. 2016;5:82. 10.12688/f1000research.7171.1 PMC4754000 26918158

[3] HealthMap: Zika outbreak. http://www.healthmap.org/zika/#timeline (2016). Accessed 21 Oct 2016.

[4] The Lancet (2016). Yellow fever: a global reckoning. Lancet..

[5] Goldani LZ (2017). Yellow fever outbreak in Brazil, 2017. Braz J Infect Dis.

[6] Frierson JG (2010). The yellow fever vaccine: a history. Yale J Biol Med..

[7] Hegde NR, Gore MM (2017). Japanese encephalitis vaccines: Immunogenicity, protective efficacy, effectiveness, and impact on the burden of disease. Hum Vaccin Immunother..

[8] Weaver SC, Barrett AD (2004). Transmission cycles, host range, evolution and emergence of arboviral disease. Nat Rev Microbiol..

[9] Liang G, Gao X, Gould EA (2015). Factors responsible for the emergence of arboviruses; strategies, challenges and limitations for their control. Emerg Microbes Infect..

[10] Hadler JL, Patel D, Nasci RS, Petersen LR, Hughes JM, Bradley K (2015). Assessment of arbovirus surveillance 13 years after introduction of West Nile virus, United States. Emerg Infect Dis.

[11] Australian Government Department of Health: Introduction to the National Notifiable Disease Surveillance System. http://www.health.gov.au/internet/main/publishing.nsf/content/cda-surveil-nndss-nndssintro.htm (2015). Accessed 31 Mar 2017.

[12] Moore CG, McLean RG, Mitchell RS, Nasci RS, Tsai TF, Calisher CH, et al. Guidelines for arbovirus surveillance programs in the United States. Fort Collins, CO: Centers for Disease Control and Prevention; 1993.

[13] Lanciotti RS, Roehrig JT, Deubel V, Smith J, Parker M, Steele K (1999). Origin of the West Nile virus responsible for an outbreak of encephalitis in the northeastern United States. Science..

[14] Eidson M, Kramer L, Stone W, Hagiwara Y, Schmit K (2001). Dead bird surveillance as an early warning system for West Nile virus. Emerg Infect Dis..

[15] Holzmann I, Agostini I, Areta JI, Ferreyra H, Beldomenico P, Di Bitetti MS (2010). Impact of yellow fever outbreaks on two howler monkey species (*Alouatta guariba clamitans* and *A. caraya*) in Misiones, Argentina. Am J Primatol..

[16] Boadle A. Dead monkey prompts Sao Paulo to step up yellow fever vaccination. https://www.reuters.com/article/us-health-yellowfever-brazil/dead-monkey-prompts-sao-paulo-to-step-up-yellow-fever-vaccination-idUSKBN1CS2UC (2017). Accessed 14 Feb 2018.

[17] Halliday JEB, Meredith AL, Knobel DL, Shaw DJ, Bronsvoort BMC, Cleaveland S (2007). A framework for evaluating animals as sentinels for infectious disease surveillance. J R Soc Interface..

[18] Hanna JN, Ritchie SA, Phillips DA, Lee JM, Hills SL, van den Hurk AF (1999). Japanese encephalitis in north Queensland, Australia, 1998. Med J Aust.

[19] Reisen WK, Lundstrom JO, Scott TW, Eldridge BF, Chiles RE, Cusack R (2000). Patterns of avian seroprevalence to western equine encephalomyelitis and Saint Louis encephalitis viruses in California, USA. J Med Entomol..

[20] Cherry B, Trock SC, Glaser A, Kramer L, Ebel GD, Glaser C (2001). Sentinel chickens as a surveillance tool for West Nile virus in New York City, 2000. Ann NY Acad Sci..

[21] Unlu I, Roy AF, Yates M, Garrett D, Bell H, Harden T (2009). Evaluation of surveillance methods for detection of West Nile virus activity in East Baton Rouge Parish, Louisiana, 2004–2006. J Am Mosq Control Assoc..

[22] Komar N (2001). West Nile virus surveillance using sentinel birds. Ann NY Acad Sci..

[23] van den Hurk AF, Ritchie SA, Johansen CA, Mackenzie JS, Smith GA (2008). Domestic pigs and Japanese encephalitis virus infection, Australia. Emerg Infect Dis..

[24] Johansen CA, Montgomery BL, Mackenzie JS, Ritchie SA (2003). Efficacies of the MosquitoMagnet™ and counterflow geometry traps in Northern Queensland, Australia. J Am Mosq Control Assoc..

[25] Hall RA, Blitvich BJ, Johansen CA, Blacksell SD. Advances in arbovirus surveillance, detection and diagnosis. J Biomed Biotechnol. 2012;2012:512969. 10.1155/2012/512969 PMC3363164 22665984

[26] Sudia WD, Lord RD, Hayes RO. Collection and processing of vertebrate specimens for arbovirus studies. Atlanta, GA: Communicable Disease Center Publication; 1970.

[27] van den Hurk AF, Hall-Mendelin S, Johansen CA, Warrilow D, Ritchie SA (2012). Evolution of mosquito-based arbovirus surveillance systems in Australia. J Biomed Biotechnol..

[28] Scott TW, Wright SA, Eldridge BF, Brown DA (2001). Cost effectiveness of three arbovirus surveillance methods in northern California. J Am Mosq Control Assoc..

[29] Gu W, Unnasch TR, Katholi CR, Lampman R, Novak RJ (2008). Fundamental issues in mosquito surveillance for arboviral transmission. Trans R Soc Trop Med Hyg..

[30] Paternina LE, Rodas JD (1604). Sampling design and mosquito trapping for surveillance of arboviral activity. Methods Mol Biol..

[31] Silver JB (2008). Mosquito ecology: field sampling methods.

[32] Bidlingmayer WL (1985). The measurement of adult mosquito population changes - some considerations. J Am Mosq Control Assoc..

[33] Johansen CA, Hall RA, van den Hurk AF, Ritchie SA, Mackenzie JS (2002). Detection and stability of Japanese encephalitis virus RNA and virus viability in dead infected mosquitoes under different storage conditions. Am J Trop Med Hyg..

[34] Blitvich BJ, Firth AE (2015). Insect-specific flaviviruses: a systematic review of their discovery, host range, mode of transmission, superinfection exclusion potential and genomic organization. Viruses..

[35] Kauffman EB, Jones SA, Dupuis AP, Ngo KA, Bernard KA, Kramer LD (2003). Virus detection protocols for west nile virus in vertebrate and mosquito specimens. J Clin Microbiol..

[36] Pyke AT, Smith IL, van den Hurk AF, Northill JA, Chuan TF, Westacott AJ (2004). Detection of Australasian flavivirus encephalitic viruses using rapid fluorogenic TaqMan RT-PCR assays. J Virol Methods..

[37] Lanciotti RS (2003). Molecular amplification assays for the detection of flaviviruses. Adv Virus Res..

[38] Sutherland GL, Nasci RS (2007). Detection of West Nile virus in large pools of mosquitoes. J Am Mosq Control Assoc..

[39] Ritchie SA, Pyke AT, Smith GA, Northill JA, Hall RA, van den Hurk AF (2003). Field evaluation of a sentinel mosquito (Diptera: Culicidae) trap system to detect Japanese encephalitis in remote Australia. J Med Entomol..

[40] Kramer LD, Chiles RE, Do TD, Fallah HM (2001). Detection of St. Louis encephalitis and western equine encephalomyelitis RNA in mosquitoes tested without maintenance of a cold chain. J Am Mosq Control Assoc..

[41] Brault AC, Fang Y, Reisen WK (2015). Multiplex qRT-PCR for the detection of western equine encephalomyelitis, St. Louis encephalitis, and West Nile viral RNA in mosquito pools (Diptera: Culicidae). J Med Entomol..

[42] Turell MJ, Spring AR, Miller MK, Cannon CE (2002). Effect of holding conditions on the detection of West Nile viral RNA by reverse transcriptase-polymerase chain reaction from mosquito (Diptera: Culicidae) pools. J Med Entomol..

[43] Ryan J, Dave K, Emmerich E, Fernandez B, Turell M, Johnson J (2003). Wicking assays for the rapid detection of West Nile and St. Louis encephalitis viral antigens in mosquitoes (Diptera: Culicidae). J Med Entomol..

[44] Hinson JM, Dave S, McMenamy SS, Dave K, Turell MJ (2015). Immuno-chromatographic wicking assay for the rapid detection of chikungunya viral antigens in mosquitoes (Diptera: Culicidae). J Med Entomol..

[45] Wanja E, Parker ZF, Odusami O, Rowland T, Dave K, Dave S (2014). Immuno-chromatographic wicking assay for the rapid detection of dengue viral antigens in mosquitoes (Diptera: Culicidae). J Med Entomol..

[46] Tan CH, Wong PS, Li MZ, Vythilingam I, Ng LC (2011). Evaluation of the dengue NS1 Ag Strip® for detection of dengue virus antigen in *Aedes aegypti* (Diptera: Culicidae). Vector Borne Zoonotic Dis..

[47] Lau SM, Chua TH, Sulaiman WY, Joanne S, Lim YA, Sekaran SD (2017). A new paradigm for *Aedes* spp. surveillance using gravid ovipositing sticky trap and NS1 antigen test kit. Parasit Vectors..

[48] Turell M, Dave K, Mayda M, Parker Z, Coleman R, Dave S (2011). Wicking assay for the rapid detection of Rift Valley fever viral antigens in mosquitoes (Diptera: Culicidae). J Med Entomol..

[49] Wanja E, Parker Z, Rowland T, Turell MJ, Clark JW, Dave K (2011). Field evaluation of a wicking assay for the rapid detection of Rift Valley fever viral antigens in mosquitoes. J Am Mosq Control Assoc..

[50] Gu W, Novak RJ (2004). Short report: detection probability of arbovirus infection in mosquito populations. Am J Trop Med Hyg..

[51] Foster WA (1995). Mosquito sugar feeding and reproductive energetics. Annu Rev Entomol..

[52] Edman JD, Strickman D, Kittayapong P, Scott TW (1992). Female *Aedes aegypti* (Diptera: Culicidae) in Thailand rarely feed on sugar. J Med Entomol..

[53] Van Handel E (1984). Metabolism of nutrients in the adult mosquito. Mosq News..

[54] Doggett S, Klowden MJ, Russel RC (2001). Are vector competence experiments competent vector experiments?. Arbovirus Res Aust..

[55] van den Hurk AF, Johnson PH, Hall-Mendelin S, Northill JA, Simmons RJ, Jansen CC (2007). Expectoration of Flaviviruses during sugar feeding by mosquitoes (Diptera: Culicidae). J Med Entomol..

[56] Hall-Mendelin S, Ritchie SA, Johansen CA, Zborowski P, Cortis G, Dandridge S (2010). Exploiting mosquito sugar feeding to detect mosquito-borne pathogens. Proc Natl Acad Sci USA..

[57] Ritchie SA, Cortis G, Paton C, Townsend M, Shroyer D, Zborowski P (2013). A simple non-powered passive trap for the collection of mosquitoes for arbovirus surveillance. J Med Entomol..

[58] Johnson BJ, Kerlin T, Hall-Mendelin S, van den Hurk AF, Cortis G, Doggett SL (2015). Development and field evaluation of the sentinel mosquito arbovirus capture kit (SMACK). Parasit Vectors..

[59] Lothrop HD, Wheeler SS, Fang Y, Reisen WK (2012). Use of scented sugar bait stations to track mosquito-borne arbovirus transmission in California. J Med Entomol..

[60] van den Hurk AF, Hall-Mendelin S, Townsend M, Kurucz N, Edwards J, Ehlers G (2014). Applications of a sugar-based surveillance system to track arboviruses in wild mosquito populations. Vector Borne Zoonotic Dis..

[61] Kurucz N, Wenham J, Hunt N, Melville L (2014). Murray Valley encephalitis virus detection using honeybait cards in the Northern Territory in 2013. Mosq Bites..

[62] State of Queensland (Queensland Health). Queensland arbovirus sentinel surveillance system report July 2016. Comunicable Diseases Branch, Prevention Division, Queensland Department of Health; 2016.

[63] Doggett S, Haniotis J, Clancy J, Webb CE, Toi C, Hueston L, et al. The New South Wales Arbovirus Surveillance and Mosquito Monitoring Program. 2014–2015 Annual Report. Westmead, Australia: Department of Medical Entomology, ICPMR, Westmead Hospital; 2015.

[64] Flies EJ, Toi C, Weinstein P, Doggett SL, Williams CR (2015). Converting mosquito surveillance to arbovirus surveillance with honey-baited nucleic acid preservation cards. Vector Borne Zoonotic Dis..

[65] Girod R, Guidez A, Carinci R, Issaly J, Gaborit P, Ferrero E (2016). Detection of chikungunya virus circulation using sugar-baited traps during a major outbreak in French Guiana. PLoS Negl Trop Dis..

[66] Eiras AE, Buhagiar TS, Ritchie SA (2014). Development of the gravid *Aedes* trap for the capture of adult female container-exploiting mosquitoes (Diptera: Culicidae). J Med Entomol..

[67] Ritchie SA, Buhagiar TS, Townsend M, Hoffmann A, Van Den Hurk AF, McMahon JL (2014). Field validation of the gravid *Aedes* trap (GAT) for collection of *Aedes aegypti* (Diptera: Culicidae). J Med Entomol..

[68] Hall-Mendelin S, Hewitson GR, Genge D, Burtonclay PJ, De Jong AJ, Pyke AT (2017). FTA® cards facilitate storage, shipment, and detection of arboviruses in infected *Aedes aegypti* collected in adult mosquito traps. Am J Trop Med Hyg..

[69] Devine TL, Venard CE, Myser WC (1965). Measurement of salivation by *Aedes aegypti* (L.) feeding on a living host. J Insect Physiol..

[70] Jones JC, Brandt E (1981). Fluid excretion by adult *Aedes aegypti* mosquitoes. J Insect Physiol..

[71] Erickson SM, Fischer K, Weil GJ, Christensen BM, Fischer PU (2009). Distribution of *Brugia malayi* larvae and DNA in vector and non-vector mosquitoes: implications for molecular diagnostics. Parasit Vectors..

[72] Pilotte N, Zaky WI, Abrams BP, Chadee DD, Williams SA (2016). A novel xenomonitoring technique using mosquito excreta/feces for the detection of filarial parasites and malaria. PLoS Negl Trop Dis..

[73] de Beaurepaire Aragão H, da Costa Lima A (1929). Sobre a transmissão do virus da febre amarella pelas fezes de mosquitos infectados. Mem Inst Oswaldo Cruz..

[74] de Beaurepaire Aragão H, da Costa Lima A (1929). Sobre o tempo necessario para que *Stegomyias* infectados excretem fezes virulentas. Mem Inst Oswaldo Cruz..

[75] de Beaurepaire Aragão H, da Costa Lima A (1929). Sobre a infecção do *M. Rhesus* pela deposição de fezes de mosquitos infectados sobre a pelle ou na conjunctiva ocular íntegras. Mem Inst Oswaldo Cruz..

[76] Fontaine A, Jiolle D, Moltini-Conclois I, Lequime S, Lambrechts L (2016). Excretion of dengue virus RNA by *Aedes aegypti* allows non-destructive monitoring of viral dissemination in individual mosquitoes. Sci Rep..

[77] Gooding RH (1972). Digestive processes of haematophagous insects. I. A literature review. Quaest Entomol..

[78] Hall-Mendelin S, Allcock R, Kresoje N, van den Hurk AF, Warrilow D (2013). Detection of arboviruses and other micro-organisms in experimentally infected mosquitoes using massively parallel sequencing. PLoS One..

[79] Naccache SN, Federman S, Veeraraghavan N, Zaharia M, Lee D, Samayoa E (2014). A cloud-compatible bioinformatics pipeline for ultrarapid pathogen identification from next-generation sequencing of clinical samples. Genome Res..

[80] Oulas A, Pavloudi C, Polymenakou P, Pavlopoulos GA, Papanikolaou N, Kotoulas G (2015). Metagenomics: tools and insights for analyzing next-generation sequencing data derived from biodiversity studies. Bioinform Biol Insights..

[81] Flygare S, Simmon K, Miller C, Qiao Y, Kennedy B, Di Sera T (2016). Taxonomer: an interactive metagenomics analysis portal for universal pathogen detection and host mRNA expression profiling. Genome Biol..

[82] Coffey LL, Page BL, Greninger AL, Herring BL, Russell RC, Doggett SL (2014). Enhanced arbovirus surveillance with deep sequencing: Identification of novel rhabdoviruses and bunyaviruses in Australian mosquitoes. Virology..

[83] Warrilow D, Watterson D, Hall RA, Davis SS, Weir R, Kurucz N (2014). A new species of mesonivirus from the Northern Territory, Australia. PLoS One..

[84] Wylie KM, Mihindukulasuriya KA, Sodergren E, Weinstock GM, Storch GA (2012). Sequence analysis of the human virome in febrile and afebrile children. PLoS One..

[85] Greninger AL, Naccache SN, Federman S, Yu G, Mbala P, Bres V (2015). Rapid metagenomic identification of viral pathogens in clinical samples by real-time nanopore sequencing analysis. Genome Med..

[86] Johnson SS, Zaikova E, Goerlitz DS, Bai Y, Tighe SW (2017). Real-time DNA sequencing in the Antarctic dry valleys using the Oxford nanopore sequencer. J Biomol Tech..

[87] Quick J, Loman NJ, Duraffour S, Simpson JT, Severi E, Cowley L (2016). Real-time, portable genome sequencing for Ebola surveillance. Nature..

[88] Quick J, Ashton P, Calus S, Chatt C, Gossain S, Hawker J (2015). Rapid draft sequencing and real-time nanopore sequencing in a hospital outbreak of *Salmonella*. Genome Biol..

[89] Batovska J, Lynch SE, Rodoni BC, Sawbridge TI, Cogan NO (2017). Metagenomic arbovirus detection using MinION nanopore sequencing. J Virol Methods..

[90] Kading RC, Biggerstaff BJ, Young G, Komar N (2014). Mosquitoes used to draw blood for arbovirus viremia determinations in small vertebrates. PLoS One..

[91] Bockarie MJ (2007). Molecular xenomonitoring of lymphatic filariasis. Am J Trop Med Hyg..

[92] Fernandez de Marco M, Brugman VA, Hernandez-Triana LM, Thorne L, Phipps LP, Nikolova NI (2016). Detection of *Theileria orientalis* in mosquito blood meals in the United Kingdom. Vet Parasitol..

[93] Rao RU, Samarasekera SD, Nagodavithana KC, Punchihewa MW, Dassanayaka TD, KDG P (2016). Programmatic use of molecular xenomonitoring at the level of evaluation units to assess persistence of lymphatic filariasis in Sri Lanka. PLoS Negl Trop Dis..

[94] Yang Y, Garver LS, Bingham KM, Hang J, Jochim RC, Davidson SA (2015). Feasibility of using the mosquito blood meal for rapid and efficient human and animal virus surveillance and discovery. Am J Trop Med Hyg..

[95] Ng TF, Willner DL, Lim YW, Schmieder R, Chau B, Nilsson C (2011). Broad surveys of DNA viral diversity obtained through viral metagenomics of mosquitoes. PLoS One..

[96] Grubaugh ND, Sharma S, Krajacich BJ, Fakoli LS, Bolay FK, Diclaro JW (2015). Xenosurveillance: a novel mosquito-based approach for examining the human-pathogen landscape. PLoS Negl Trop Dis..

[97] Brinkmann A, Nitsche A, Kohl C. Viral metagenomics on blood-feeding arthropods as a tool for human disease surveillance. Int J Mol Sci. 2016;17:pii:E1743. 10.3390/ijms17101743 PMC5085771 27775568

[98] Barbazan P, Thitithanyanont A, Misse D, Dubot A, Bosc P, Luangsri N (2008). Detection of H5N1 avian influenza virus from mosquitoes collected in an infected poultry farm in Thailand. Vector Borne Zoonotic Dis..

[99] Farid HA, Morsy ZS, Helmy H, Ramzy RM, El Setouhy M, Weil GJA (2007). critical appraisal of molecular xenomonitoring as a tool for assessing progress toward elimination of lymphatic filariasis. Am J Trop Med Hyg..

[100] Blow JA, Turell MJ, Walker ED, Silverman AL (2002). Post-bloodmeal diuretic shedding of hepatitis B virus by mosquitoes (Diptera: Culicidae). J Med Entomol..

[101] Eiras AE, Resende MC (2009). Preliminary evaluation of the 'Dengue-MI' technology for *Aedes aegypti* monitoring and control. Cad Saude Publica..

[102] Palmer JRB, Oltra A, Collantes F, Delgado JA, Lucientes J, Delacour S (2017). Citizen science provides a reliable and scalable tool to track disease-carrying mosquitoes. Nat Commun..

[103] The GLOBE Program: GLOBE Observer. https://observer.globe.gov/ (2018). Accessed 4 Jan 2018.

[104] Jordan RC, Sorensen AE, Ladeau S (2017). Citizen science as a tool for mosquito control. J Am Mosq Control Assoc..

[105] Mukundarajan H, Hol FJH, Castillo EA, Newby C, Prakash M. Using mobile phones as acoustic sensors for high-throughput mosquito surveillance. eLife. 2017;6:pii:e27854. 10.7554/eLife.27854 PMC5663474 29087296

[106] Yetisen AK, Akram MS, Lowe CR (2013). Paper-based microfluidic point-of-care diagnostic devices. Lab Chip..

[107] Ramnani P, Saucedo NM, Mulchandani A (2016). Carbon nanomaterial-based electrochemical biosensors for label-free sensing of environmental pollutants. Chemosphere..

[108] Whitesides GM (2006). The origins and the future of microfluidics. Nature..

[109] Sharma S, Zapatero-Rodriguez J, Estrela P, O'Kennedy R (2015). Point-of-care diagnostics in low resource settings: present status and future role of microfluidics. Biosensors (Basel)..

[110] Weng CH, Huang TB, Huang CC, Yeh CS, Lei HY, Lee GB (2011). A suction-type microfluidic immunosensing chip for rapid detection of the dengue virus. Biomed Microdevices..

[111] Zhang Y, Bai J, Ying JY (2015). A stacking flow immunoassay for the detection of dengue-specific immunoglobulins in salivary fluid. Lab Chip..

[112] Lee YF, Lien KY, Lei HY, Lee GB (2009). An integrated microfluidic system for rapid diagnosis of dengue virus infection. Biosens Bioelectron..

[113] Tan JJ, Capozzoli M, Sato M, Watthanaworawit W, Ling CL, Mauduit M (2014). An integrated lab-on-chip for rapid identification and simultaneous differentiation of tropical pathogens. PLoS Negl Trop Dis..

[114] Wasik D, Mulchandani A, Yates MV (2018). Point-of-use nanobiosensor for detection of dengue virus NS1 antigen in adult *Aedes aegypti*: a potential tool for improved dengue surveillance. Anal Chem..

[115] Liu C, Mauk MG, Hart R, Bonizzoni M, Yan G, Bau HH (2012). A low-cost microfluidic chip for rapid genotyping of malaria-transmitting mosquitoes. PLoS One..

[116] Gardy J, Loman NJ, Rambaut A (2015). Real-time digital pathogen surveillance - the time is now. Genome Biol..

[117] Meyer Steiger DB, Ritchie SA, Laurance SG (2014). Overcoming the challenges of mosquito (Diptera: Culicidae) sampling in remote localities: a comparison of CO_2_ attractants on mosquito communities in three tropical forest habitats. J Med Entomol..

[118] Inglis TJ, Bradbury RS, McInnes RL, Frances SP, Merritt AJ, Levy A (2016). Deployable molecular detection of arboviruses in the Australian outback. Am J Trop Med Hyg..

[119] Kwan JL, Kluh S, Madon MB, Nguyen DV, Barker CM, Reisen WK (2010). Sentinel chicken seroconversions track tangential transmission of West Nile virus to humans in the greater Los Angeles area of California. Am J Trop Med Hyg..

[120] Buckley A, Dawson A, Gould EA (2006). Detection of seroconversion to West Nile virus. Usutu virus and Sindbis virus in UK sentinel chickens. Virol J..

[121] Day JF, Winner R, Parsons RE, Zhang JT (1991). Distribution of St. Louis encephalitis viral antibody in sentinel chickens maintained in Sarasota County, Florida: 1978–1988. J Med Entomol..

[122] Broom AK, Azuolas J, Hueston L, Mackenzie JS, Melville L, Smith DW (2001). Australian encephalitis: sentinel chicken surveillance programme. Commun Dis Intell Q Rep..

[123] Chuang TW, Knepper RG, Stanuszek WW, Walker ED, Wilson ML (2011). Temporal and spatial patterns of West Nile virus transmission in Saginaw County, Michigan, 2003–2006. J Med Entomol..

[124] Morris CD, Baker WG, Stark L, Burgess J, Lewis AL (1994). Comparison of chickens and pheasants as sentinels for eastern equine encephalitis and St. Louis encephalitis viruses in Florida. J Am Mosq Control Assoc.

[125] Maeda O, Takenokuma K, Karoji Y, Kuroda A, Sasaki O, Karaki T (1978). Epidemiological studies on Japanese encephalitis in Kyoto city area, Japan. Jpn J Med Sci Biol.

[126] Shield J, Hanna J, Phillips D (1996). Reappearance of the Japanese encephalitis virus in the Torres Strait, 1996. Commun Dis Intell.

[127] Nitatpattana N, Le Flohic G, Thongchai P, Nakgoi K, Palaboodeewat S, Khin M (2011). Elevated Japanese encephalitis virus activity monitored by domestic sentinel piglets in Thailand. Vector Borne Zoonotic Dis..

[128] Resnick MP, Grunenwald P, Blackmar D, Hailey C, Bueno R, Murray KO (2008). Juvenile dogs as potential sentinels for West Nile virus surveillance. Zoonoses Public Health..

[129] Davoust B, Leparc-Goffart I, Demoncheaux JP, Tine R, Diarra M, Trombini G (2014). Serologic surveillance for West Nile virus in dogs, Africa. Emerg Infect Dis..

[130] Shimoda H, Ohno Y, Mochizuki M, Iwata H, Okuda M, Maeda K (2010). Dogs as sentinels for human infection with Japanese encephalitis virus. Emerg Infect Dis..

[131] Shimoda H, Inthong N, Noguchi K, Terada Y, Nagao Y, Shimojima M (2013). Development and application of an indirect enzyme-linked immunosorbent assay for serological survey of Japanese encephalitis virus infection in dogs. J Virol Methods..

[132] Lichoti JK, Kihara A, Oriko AA, Okutoyi LA, Wauna JO, Tchouassi DP (2014). Detection of Rift Valley fever virus interepidemic activity in some hotspot areas of Kenya by sentinel animal surveillance, 2009–2012. Vet Med Int..

[133] Zeller HG, Fontenille D, Traore-Lamizana M, Thiongane Y, Digoutte JP (1997). Enzootic activity of Rift Valley fever virus in Senegal. Am J Trop Med Hyg..

[134] Al-Qabati AG, Al-Afaleq AI (2010). Cross-Sectional, Longitudinal and prospective epidemiological studies of Rift Valley fever in Al-Hasa oasis, Saudi Arabia. J Anim Vet Adv..

[135] Gard GP, Shorthose JE, Weir RP, Walsh SJ, Melville LF (1988). Arboviruses recovered from sentinel livestock in northern Australia. Vet Microbiol..

[136] St. George TD (1980). A sentinel herd system for the study of arbovirus infections in Australia and Papua-New Guinea. Vet Res Commun..

[137] Kato T, Shirafuji H, Tanaka S, Sato M, Yamakawa M, Tsuda T (2016). Bovine arboviruses in *Culicoides* biting midges and sentinel cattle in southern Japan from 2003 to 2013. Transbound Emerg Dis..

[138] Monath TP, Sabattini MS, Pauli R, Daffner JF, Mitchell CJ, Bowen GS, et al. Arbovirus investigations in Argentina, 1977–1980. IV. Serologic surveys and sentinel equine program. Am J Trop Med Hyg. 1985;34:966–975. 2863991

[139] Mattar S, Komar N, Young G, Alvarez J, Gonzalez M (2011). Seroconversion for West Nile and St. Louis encephalitis viruses among sentinel horses in Colombia. Mem Inst Oswaldo Cruz..

[140] Ventura AK, Ehrenkranz NJ (1975). Detection of Venezuelan equine encephalitis virus in rural communities of Southern Florida by exposure of sentinel hamsters. Am J Trop Med Hyg..

[141] Scherer WF, Dickerman RW, Ordonez JV, Seymour C, Kramer LD, Jahrling PB (1976). Ecologic studies of Venezuelan encephalitis virus and isolations of Nepuyo and Patois viruses during 1968–1973 at a marsh habitat near the epicenter of the 1969 outbreak in Guatemala. Am J Trop Med Hyg..

[142] Walder R, Suarez OM (1976). Studies of arboviruses in southwestern Venezuela: I. Isolations of Venezuelan and eastern equine encephalitis viruses from sentinel hamsters in the Catatumbo region. Int J Epidemiol..

[143] Calisher CH, Gutierrez E, Francy DB, Alava A, Muth DJ, Lazuick JS (1983). Identification of hitherto unrecognized arboviruses from Ecuador: members of serogroups B, C, Bunyamwera, Patois, and Minatitlan. Am J Trop Med Hyg..

[144] Batista PM, Andreotti R, Chiang JO, Ferreira MS, Vasconcelos PF (2012). Seroepidemiological monitoring in sentinel animals and vectors as part of arbovirus surveillance in the state of Mato Grosso do Sul, Brazil. Rev Soc Bras Med Trop..

[145] Morales MA, Fabbri CM, Zunino GE, Kowalewski MM, Luppo VC, Enria DA (2017). Detection of the mosquito-borne flaviviruses, West Nile, dengue, Saint Louis encephalitis, Ilheus, bussuquara, and yellow fever in free-ranging black howlers (*Alouatta caraya*) of Northeastern Argentina. PLoS Negl Trop Dis..

[146] de Almeida MA, Dos Santos E, da Cruz Cardoso J, da Fonseca DF, Noll CA, Silveira VR (2012). Yellow fever outbreak affecting *Alouatta* populations in southern Brazil (Rio Grande do Sul State), 2008–2009. Am J Primatol..

[147] Bensabath G, Shope RE, de Andrade AH, de Souza AP (1966). Recuperación de virus amarílico, procedente de un mono centinela, en las cercanias de Belem, Brasil. Bol Oficina Sanit Panam.

[148] Henderson BE, Metselaar D, Kirya GB, Timms GL (1970). Investigations into yellow fever virus and other arboviruses in the northern regions of Kenya. Bull World Health Organ..

[149] Krockel U, Rose A, Eiras AE, Geier M (2006). New tools for surveillance of adult yellow fever mosquitoes: comparison of trap catches with human landing rates in an urban environment. J Am Mosq Control Assoc..

[150] Sudia WD, Chamberlain RW (1962). Battery-operated light trap, an improved model. Mosq News.

[151] Rohe DL, Fall RPA (1979). miniature battery powered CO_2_ baited light trap for mosquito borne encephalitis surveillance. Bull Soc Vector Ecol..

[152] Kline DL (2002). Evaluation of various models of propane-powered mosquito traps. J Vector Ecol..

[153] Mitchell CJ, Darsie RF, Monath TP, Sabattini MS, Daffner J (1985). The use of an animal-baited net trap for collecting mosquitoes during western equine encephalitis investigations in Argentina. J Am Mosq Control Assoc..

[154] Jansen CC, Zborowski P, Ritchie SA, van den Hurk AF (2009). Efficacy of bird-baited traps placed at different heights for collecting ornithophilic mosquitoes in eastern Queensland, Australia. Aust J Entomol..

[155] Darbro JM, Harrington LC (2006). Bird-baited traps for surveillance of West Nile mosquito vectors: effect of bird species, trap height, and mosquito escape rates. J Med Entomol..

[156] Schoeler GB, Schleich SS, Manweiler SA, Lopez Sifuentes V (2004). Evaluation of surveillance devices for monitoring *Aedes aegypti* in an urban area of northeastern Peru. J Am Mosq Control Assoc..

[157] Vazquez-Prokopec GM, Galvin WA, Kelly R, Kitron U (2009). A new, cost-effective, battery-powered aspirator for adult mosquito collections. J Med Entomol..

[158] Gusciora WR. The resting box technique for the sampling of *Culiseta melanura* (Coquillet). Proc N J Mosq Exterm Assoc. 1971:122–5.

[159] Komar N, Pollack RJ, Spielman A. A nestable fiber pot for sampling resting mosquitoes. J Am Mosq Control Assoc. 1995;11:463–467. 8825509

[160] Panella NA, Crockett RJ, Biggerstaff BJ, Komar N (2011). The centers for disease control and prevention resting trap: a novel device for collecting resting mosquitoes. J Am Mosq Control Assoc..

[161] Ordoñez-Gonzalez JG, Mercado-Hernandez R, Flores-Suarez AE, Fernandez-Salas I (2001). The use of sticky ovitraps to estimate dispersal of *Aedes aegypti* in northeastern Mexico. J Am Mosq Control Assoc..

[162] Ritchie SA, Long S, Hart A, Webb CE, Russell RC (2003). An autocidal sticky ovitrap for sampling container-breeding mosquitoes. J Am Mosq Control Assoc..

[163] Mackay AJ, Amador M, Barrera R (2013). An improved autocidal gravid ovitrap for the control and surveillance of *Aedes aegypti*. Parasit Vectors..

[164] Reiter PA (1983). portable battery-powered trap for collecting gravid *Culex* mosquitoes. Mosq News..

